# Cost-Effectiveness of Intermittent Preventive Treatment of Malaria in Pregnancy in Southern Mozambique

**DOI:** 10.1371/journal.pone.0013407

**Published:** 2010-10-15

**Authors:** Elisa Sicuri, Azucena Bardají, Tacilta Nhampossa, Maria Maixenchs, Ariel Nhacolo, Delino Nhalungo, Pedro L. Alonso, Clara Menéndez

**Affiliations:** 1 Barcelona Centre for International Health Research (CRESIB), Hospital Clínic/IDIBAPS, Universitat de Barcelona, Barcelona, Spain; 2 CIBER Epidemiología y Salud Pública (CIBERESP), Barcelona, Spain; 3 Centro de Investigação em Saúde da Manhiça (CISM), Maputo, Mozambique; 4 Direcção Nacional de Saúde/Instituto Nacional de Saúde, Ministerio de Saúde, Maputo, Mozambique; Menzies School of Health Research, Australia

## Abstract

**Background:**

Malaria in pregnancy is a public health problem for endemic countries. Economic evaluations of malaria preventive strategies in pregnancy are needed to guide health policies.

**Methods and Findings:**

This analysis was carried out in the context of a trial of malaria intermittent preventive treatment in pregnancy with sulphadoxine-pyrimethamine (IPTp-SP), where both intervention groups received an insecticide treated net through the antenatal clinic (ANC) in Mozambique. The cost-effectiveness of IPTp-SP on maternal clinical malaria and neonatal survival was estimated. Correlation and threshold analyses were undertaken to assess the main factors affecting the economic outcomes and the cut-off values beyond which the intervention is no longer cost-effective. In 2007 US$, the incremental cost-effectiveness ratio (ICER) for maternal malaria was 41.46 US$ (95% CI 20.5, 96.7) per disability-adjusted life-year (DALY) averted. The ICER per DALY averted due to the reduction in neonatal mortality was 1.08 US$ (95% CI 0.43, 3.48). The ICER including both the effect on the mother and on the newborn was 1.02 US$ (95% CI 0.42, 3.21) per DALY averted. Efficacy was the main factor affecting the economic evaluation of IPTp-SP. The intervention remained cost-effective with an increase in drug cost per dose up to 11 times in the case of maternal malaria and 183 times in the case of neonatal mortality.

**Conclusions:**

IPTp-SP was highly cost-effective for both prevention of maternal malaria and reduction of neonatal mortality in Mozambique. These findings are likely to hold for other settings where IPTp-SP is implemented through ANC visits. The intervention remained cost-effective even with a significant increase in drug and other intervention costs. Improvements in the protective efficacy of the intervention would increase its cost-effectiveness. Provision of IPTp with a more effective, although more expensive drug than SP may still remain a cost-effective public health measure to prevent malaria in pregnancy.

**Trial Registration:**

ClinicalTrials.gov NCT00209781

## Introduction

Malaria during pregnancy can result in negative outcomes in maternal and child health [1], [2]. For this reason the World Health Organization (WHO) currently recommends the administration of intermittent preventive treatment (IPTp) with sulfadoxine-pyrimethamine (SP) and the provision of insecticide treated nets (ITNs) [3]. Interestingly, although IPTp-SP has been recommended for the last 12 years [4] there is still little and incomplete information on the economic evaluation of this strategy. All previously published economic evaluations of IPTp-SP have used surrogate indicators of infant mortality, such as low birth weight and parasitemia or placental malaria as surrogate indicators of maternal morbidity and mortality to calculate disability adjusted life years (DALYs) [5]–[9]. Only two cost-effectiveness analysis of IPTp-SP have been carried out alongside intervention studies [5], [6]. Amid the increasing attention given to malaria eradication [10], [11], there is still a need to conduct economic evaluations of control strategies in general and specifically in pregnancy, to inform health policy decision making [12].

IPTp involves giving at least two treatment courses of SP to pregnant women from the second trimester onwards at least one month apart. The increasing resistance of the parasite to SP leads to the need of evaluating safety and efficacy of new drugs for IPTp [13]–[15]. Increasing the effectiveness of malaria preventive interventions in pregnancy would require available safe and more efficacious drugs for IPTp as well as improving antenatal clinic (ANC) attendance and the quality of ANC services [16], [17]. However, new strategies for effectiveness improvement are likely to entail extra costs to the health system. As a consequence, the economic evaluation of IPTp-SP should also include the estimation of the cut-off levels of the intervention costs beyond which the strategy ceases to be cost-effective under different epidemiological conditions, levels of efficacy and factors that may limit effectiveness.

In order to facilitate the decision making process of malaria control in pregnancy, we carried out a cost-effectiveness analysis of IPTp with SP based on efficacy results of a trial in which the intervention was tested against IPTp with placebo and women in both trial arms received an ITN. The provision of IPTp and ITNs was implemented through the ANC of a district hospital in Southern Mozambique. This is the first cost-effectiveness analysis of IPTp-SP to consider its incremental effect in addition to ITNs and to evaluate its consequences on clinical maternal malaria and on neonatal mortality. The main factors affecting the cost-effectiveness of the intervention were evaluated, as well as the cut-off points beyond which IPTp-SP is no longer cost-effective.

## Methods

### Study area and population

The study was undertaken at the Centro de Investigação em Saúde da Manhiça (CISM) in Manhiça, Maputo Province, Southern Mozambique. The CISM carries out a Demographic Surveillance System (DSS) in the Manhiça study area, which includes a population of 80.000 inhabitants. Adjacent to the CISM is the Manhiça District Hospital (MDH), a 110 bed health facility.

The whole Manhiça District has an estimated population of about 130.000 inhabitants. The main local economic activity is subsistence farming and some workers are employed in two sugar and fruit processing factories. An increasing number of small and medium traders have established their activity along the road Maputo-Beira. The two main towns are Manhiça and Xinavane but most of the population live in small dispersed hamlets. Malaria transmission in the area is perennial with some seasonality and the entomological inoculation rate for the year 2002 was 38 infective bites per person per year [18]. Geographical and demographic characteristics have been described elsewhere [19], [20].

ANC attendance is high in the study area with more than 95% of the women attending the ANC at least once during pregnancy (Nhacolo, personal communication).

Bed net use is limited in the area. Around 40% of the households' representatives interviewed during a recent study conducted among the DSS population replied to own at least one (non-impregnated) bed net. Of those, less than 40% referred that they had slept under the bed net during the previous rainy season [21].

### Study design

#### Effects

This economic evaluation was done in the context of a randomized, placebo-controlled trial of two doses of SP as IPTp. Pregnant women were enrolled at the ANC and individually randomised to receive placebo or SP. All participant women received a long-lasting ITN (LLITN) as part of the study. The main results of the trial showed that IPTp-SP was efficacious in reducing clinical malaria during pregnancy [Protective Efficacy (PE) 40% (95% CI 7.4%, 61.2%); p = 0.020] and neonatal mortality [PE 61.3% (95% CI 7.4%, 83.8%); p = 0.024] [22], [23]. The intervention was safe and well tolerated as shown in other studies [24]–[26]. More than 80% of the women reported having used the ITN during pregnancy and 62% after delivery (Bardají et al, unpublished). This economic evaluation was included in the trial protocol. The trial protocol was approved by the National Mozambican Ethics Review Committee, and the Hospital Clinic of Barcelona Ethics Review Committee.

### Costs

#### IPTp intervention costs

As variable costs of the intervention, the value of the nurses' time to administer the intervention, as well as drug costs were considered. During real time observations of routine ANC visits at the MDH, it was estimated that personnel costs of administering three tablets of SP to a pregnant woman represented three minutes of a nurse's full workload, i.e. 0.70% of her daily wage. International SP drug prices were used. Drug prices were increased by 10% to include shipping costs [27]. Fixed costs were represented by the cost of training health personnel on the administration of IPTp-SP. Because of the similarities between the two types of preventive interventions, training costs were assumed to be equal to those estimated in a previous study on Intermittent Preventive Treatment of malaria in infants (IPTi) [28]. Other non-recurrent components considered in the IPTi implementation cost estimate, such as policy change and sensitisation, strategy management and development of behaviour change communication materials, were not included in this study because they were considered negligible for the implementation of IPTp-SP in Mozambique [29]. Intervention costs for a target of 1000 pregnant women receiving IPTp-SP at the ANC was equal to the unit fixed costs component multiplied by 2000 (2 doses*1000 women) plus the unit variable costs component multiplied by the actual number of doses delivered. The number of IPTp doses delivered was calculated considering that of the target of 1000 pregnant women, 98% attend the ANC at least once and between 85% and 92% at least twice during pregnancy [30].

#### Costs of malaria treatment during pregnancy

Health system costs for the treatment of a malaria episode included admission costs at the maternity ward in the case of inpatients, or the cost of attendance and treatment in the case of outpatients [31]. Unit costs of an admission at the maternity ward (occupied bed/day) and of an external consultation included all recurrent components and excluded capital costs. Recurrent costs consisted of personnel, medical, surgical and laboratory supplies among other recurrent costs. Unit costs were updated from the year 2000 to the year 2007 using the average annual rate inflation correction factor and validated during interviews with the administrative staff of the MDH [32]. Drug costs were added to this estimate. Quinine was the antimalarial drug administered to admitted pregnant women and to outpatient pregnant women in the first trimester of gestation. The combination of SP and artesunate was administered to outpatient pregnant women who were over the first trimester of pregnancy. Drug costs for malaria treatment are mostly sustained by the National Health System in Mozambique, and patients only pay a small fee when attending government health facilities. Total admission costs were calculated by multiplying the daily unit cost of admission at the maternity ward by the average number of admission days, which were estimated through the household cost data collection outlined below.

IPTp-SP net intervention costs for 1000 pregnant women receiving the intervention were calculated as difference between intervention costs and health system costs for the treatment of malaria episodes averted.

Household costs of malaria treatment during pregnancy were collected from July 2007 to May 2008 through standardized questionnaires administered to pregnant women when leaving the maternity ward of the MDH after being admitted with an episode of malaria (*n* = 34), or after attending the outpatient clinic with a diagnosis of malaria (*n* = 66). Only those women who signed a written informed consent had the questionnaire administered. Direct household costs included transportation to and from the hospital, food and other expenses. Estimated indirect costs included reductions in paid and unpaid production (income and welfare losses) that women incurred due to their illness. Self-reported time cost was used when women were able to estimate the amount of money lost because of the break from their routine activity. The monetary value of self-reported time lost was estimated according to the minimum wage in force in Mozambique if women could not provide this estimate [33].

Household costs data collected were double entered in FoxPro (Microsoft Corp., Seattle, WA, USA) and data cleaning and analysis were performed with STATA 9 (Stata Corporation, College Station, TX, USA). All cost data are presented in United States Dollars (US$) for the year 2007.

#### Sample size for household costs survey

Sample size for the minimum number of women to be administered with a questionnaire was calculated using this formula: *n* = *(Z^2^pq/e^2^)/{1 + [(Z^2^pq/e^2^)−1]/N*} [34]. In the formula *Z* (confidence level) was assumed to be 95%; *e (*level of precision) was assumed to be 7%; *p* (household costs variability) was assumed to be 30%; *q* = *1−p*; *N* (population of reference) was represented by 200 pregnant women with malaria registered at the maternity ward of the MDH during the year 2006. *n* resulted to be equal to 91. However, information on 100 women was collected. Thirty percent of the sample was assumed to be admission cases [35].

### Cost effectiveness analysis

All model inputs were expressed as probability distributions (Table 1). Bootstrapping techniques were used to calculate distribution ranges in the case of household costs after assessing the non-normality of cost distributions through Shapiro-Wilk tests [36], [37]. Ranges were derived from different published sources when individual data were not available or assumed to be ±25% of the estimated mean value in cases where there was no information on plausible ranges in the literature [38].

**Table 1:** Input variables of the probabilistic cost-effectiveness analysis of IPTp-SP^a^.

Probaility input variables	Type of probability distribution^b^	Low estimate	Best estimate	High estimate	Sources
**IPTp intervention costs per dose delivered** c					
Drug	Triangular	0.06	0.07	0.13	[27]
Personnel	Triangular	0.08	0.10	0.13	Observational study
Training	Triangular	0.04	0.05	0.07	[28]
**Antenatal clinic attendance**					
At least once	Point estimate		0.98		[30]
At least twice	Uniform	0.85		0.92	[30]
**Epidemiological inputs and efficacy of IPTp-SP** a **on maternal health**					
Protective efficacy of IPTp-SP^a^	Triangular	0.074	0.40	0.61	[22]
Malaria incidence^d^	Triangular	0.26	0.35	0.44	[22]
Proportion of malaria cases seeking care^e^	Uniform	0.40		0.60	[53]
Proportion of malaria cases that are hospitalized^f^	Triangular	0.03	0.04	0.05	[22]
Case Fatality Rate	Triangular	0.0026	0.0033	0.0045	Estimate
**Household costs for malaria treatment of pregnant women** c					
***Inpatients***					
Direct^g^	Triangular	−12.16	5.10	12.55	Survey
Indirect^g^	Triangular	−6.38	5.01	9.02	Survey
***Outpatients***					
Direct	Triangular	0.01	0.61	1.21	Survey
Indirect	Triangular	1.08	1.49	1.91	Survey
**Health system costs for malaria treatment of pregnant women** c					
***Inpatients***					
Drug^h^	Triangular	1.28	1.52	2.87	[27]
Inpatient average cost per admission/day	Triangular	29.41	39.21	49.01	[31]
***Outpatients***					
Drug^i^	Triangular	1.91	3.97	4.22	[27]
Visits	Triangular	0.67	0.90	1.10	[31]
**Efficacy of IPTp-SP** a **on neonatal mortality**					
Number of neonatal deaths averted due to IPTp-SP^a^	Triangular	0.00	11.00	22.00	[23]
Reduction of neonatal deaths per 1000 mothers receiving SP^l^	Triangular	4.96	22.22	39.47	[23]

a Intermittent preventive treatment of malaria in pregnancy with sulphadoxine-pyrimethamine.

b Tringular distribution was chosen to be consistent with previous similar studies [53], [58].

c in US$ 2007.

d Rate per person-year at risk in the placebo group.

e It indicates the proportion of pregnant women with symptoms of malaria who seek formal health care. The values of the uniform distribution are adapted from Hutton et al [53]

f It is assumed that severe cases  =  hospitalized cases

g The left limit of the confidence interval is negative due to bootstrapping.

h Drug costs for inpatients refers to intravenous quinine.

i Drug costs for outpatients refers to artesunate plus SP.

l Seven newborns died during the first 28 days of life for each 495 pregnant women receiving SP and 18 newborns died for 493 pregnant women receiving placebo. Reduction of deaths per 1000 mothers receiving SP is equal to [number of deaths averted/number of mothers in SP group]*1000.

For a reference population of 1000 pregnant women, incremental cost-effectiveness ratios (ICERs) were calculated by dividing IPTp-SP intervention costs by the number of Disability-Adjusted Life Years (DALYs) averted due to the reduction in maternal clinical malaria, or in neonatal deaths, respectively. Therefore, ICERs included gross intervention costs and excluded saving from fewer cases of maternal malaria consequent to the administration of IPTp-SP. DALYs averted were calculated by multiplying DALYs lost from maternal malaria or neonatal deaths, times the effectiveness of the intervention on these two outcomes. In this calculation, age weighting (β = 0.04) and 3% discount rate were taken into account, and DALYs were based on standard measures of disease duration (the duration of a non-complicated malaria episode effectively treated was assumed to be on average 3.5 days, corresponding to 0.01 years) and impact on the quality of life (disability weight for an episode of malaria in adults was assumed to be 0.172) [39]. The effectiveness of the intervention with respect to the number of maternal malaria episodes and of neonatal deaths averted was calculated by multiplying the efficacy of the intervention by the factors that may affect its implementation, such as ANC attendance. No effect of morbidity due to low birth weight or other possible effects of malaria in pregnancy have been included in DALYs calculation.

The analysis was done separated for the mother and for the newborn in order to highlight the cost-effectiveness of IPTp-SP on the reduction of neonatal mortality. For the fist time such reduction was directly associated with the intervention instead of being mediated by a decrease in low birth weight prevalence or an increase in average weight at birth [23]. However, an aggregate ICER was also calculated by dividing intervention costs by the combined DALYs averted for maternal malaria and neonatal deaths.

#### Cost-effectiveness of IPTp-SP on maternal malaria

The number of malaria episodes averted per 1000 pregnant women receiving IPTp-SP was calculated by multiplying 1000 by the PE of the intervention, the malaria incidence [as rate per person-year at risk (PYAR) in the placebo group] and the percentage of ANC attendance, at least twice, during pregnancy [30]. DALYs averted were calculated by multiplying the number of DALYs lost due to the disease by the reduction of malaria morbidity and mortality as a result of the intervention. DALYs lost were calculated according to life expectancy in Mozambique of a 24 years old woman (average age of women enrolled in the trial), which was of 40.4 years in the year 2006 and according to an estimated average case fatality rate for malaria in pregnancy of 0.33% [40], [41]. This estimate was based on the assumption that of 25 million pregnancies in Sub-Saharan Africa being at risk of malaria every year, 30% would be infected with malaria [42], [43]. Since the number of maternal deaths due to malaria every year is estimated to be approximately of 25.000, it was calculated that 0.33% of infected women would die because of the disease [25.000/(25.000.000*30%)*100 = 0.33%].

Health system savings were calculated by multiplying inpatient and outpatient health facility costs for malaria treatment by inpatient and outpatient episodes averted, respectively. Inpatient malaria episodes averted were the total number of malaria episodes averted times the proportion of cases that were admitted to hospital with malaria. Outpatient malaria episodes averted were the result of the total number of malaria episodes averted times the percentage of presumptive malaria cases seeking care at governmental facilities.

#### Cost-effectiveness of IPTp-SP on neonatal mortality

DALYs averted were calculated by multiplying the number of neonatal deaths averted by the number of DALYs lost due to neonatal mortality. The number of neonatal deaths averted per 1000 pregnant women receiving IPTp-SP was calculated by multiplying 1000 by ANC attendance, and by the reduction of deaths resulting from the intervention [44]. DALYs lost were calculated based on life expectancy at birth in the DSS area of Manhiça, which was of 46.3 years in 2007 (Nhacolo, personal communication).

ICERs are presented as acceptability curves [45], [46]. Acceptability curves allow the graphic representation of the probability that IPTp-SP is cost-effective (Y axis) according to the different investment levels in which policy makers may be able or willing to pay for each DALY averted (X axis).

### Uncertainty of parameters used in the analysis

Probabilistic cost-effectiveness analysis was undertaken through Monte Carlo simulations using @Risk (version 5.0) add-in tool to Microsoft Excel© (Palisade Corporation, Ithaca, NY, USA).

The main factors affecting the cost-effectiveness of the intervention were identified in the estimated model by calculating Spearman's rank correlation coefficients as a measure of the magnitude of the association between each variable and the ICERs. A threshold analysis of the cost-effectiveness of IPTp-SP was performed to estimate cut-off points beyond which the prevention is no longer cost-effective. The threshold level of the ICERs used to define the intervention as cost-effective was 129 US$ per DALY averted, while 36 US$ per DALY averted was the threshold used to define the intervention as highly cost-effective. These threshold figures were based on previously accepted 1993 and 1996 World Bank definitions and inflated to their 2007 equivalent: 129 and 36 US$ for the year 2007 correspond to 100 and 25 US$ for the year 1993, respectively [47]-[49]. The cost-effective intervention threshold was conservatively set at 100 instead of 150 US$ per DALY averted. Probabilistic threshold analysis was performed on SP price, other intervention costs, case fatality rate, malaria incidence, protective efficacy and antenatal clinic attendance. Furthermore, a one-way sensitivity analysis was undertaken on SP price.

## Results

Table 2 shows the results of the cost effectiveness analysis (CEA). Intervention costs of delivering two doses of IPTp-SP to 1000 pregnant women through the ANC were 435.79 US$ (CI 95% 371.80, 508.00).

**Table 2:** Cost-effectiveness analysis of IPTp-SP^a^ for 1000 pregnant women^b^.

Intervention costs^c^	435.79 (371.80, 508.00)
**Treatment savings due to the efficacy of IPTp-SP** a **on clinical malaria** c	
*Health system treatment savings*	422.74 (152.00, 718.00)
Outpatient	239.91 (84.00, 432.00)
Inpatient	182.82 (66.30, 308.00)
*Households' outpatient treatment savings*	117.69 (40.50, 212.70)
Direct	33.89 (6.10, 77.20)
Indirect	83.79 (29.60, 148.30)
*Households' admission treatment savings*	19.64 (−39.30, 81.00)
Direct	8.20 (−42.80, 55.80)
Indirect	11.44 (−20.50, 42.70)
**Net intervention costs** c **on clinical malaria**	
Intervention costs – health system treatment savings	13.17 (−292.00, 290.00)
**Incremental cost effectiveness ratio on clinical malaria**	
Intervention costs/ Number DALYs averted^d^	41.46 (20.50, 96.70)
**Effectiveness of IPTp-SP** a **on clinical malaria**	
Total number of episodes averted^e^	112.00 (42.00, 182.00)
Number of outpatient episodes averted	56.12 (20.50, 95.50)
Number of inpatient episodes averted	4.49 (1.63, 7.56)
Number of maternal deaths averted	0.39 (0.143, 0.661)
Number of DALYs^d^ averted	12.20 (4.59, 20.81)
**Incremental cost effectiveness ratio on neonatal mortality**	
Intervention costs/ Number DALYs averted^d^	1.08 (0.43, 3.48)
**Effectiveness of IPTp-SP** a **on neonatal mortality**	
Number of neonatal deaths averted	18.93 (4.39, 33.85)
Number of DALYs averted^d^	555.21 (129.00, 992.00)
**Combined analysis**	
Intervention costs/Number of DALYs averted^d^	1.02 (0.42, 3.21)
Number of DALYs averted^d^	570.95 (236.00, 908.00)

a Intermittent preventive treatment of malaria in pregnancy with sulphadoxine-pyrimethamine.

b 95% confidence intervals in brackets.

c in US$ 2007.

d Disability-adjusted life years.

e Total number of episodes averted is theoretical and relies on the assumption that formal treatment is sought for any case of suspected malaria. The total number is higher than the sum of inpatients and outpatients episodes averted because number of outpatient episodes considers that only a proportion of pregnant women with symptoms of malaria, actually, seeks formal treatment.

### Cost-effectiveness of IPTp on maternal health

**Figure 1:**
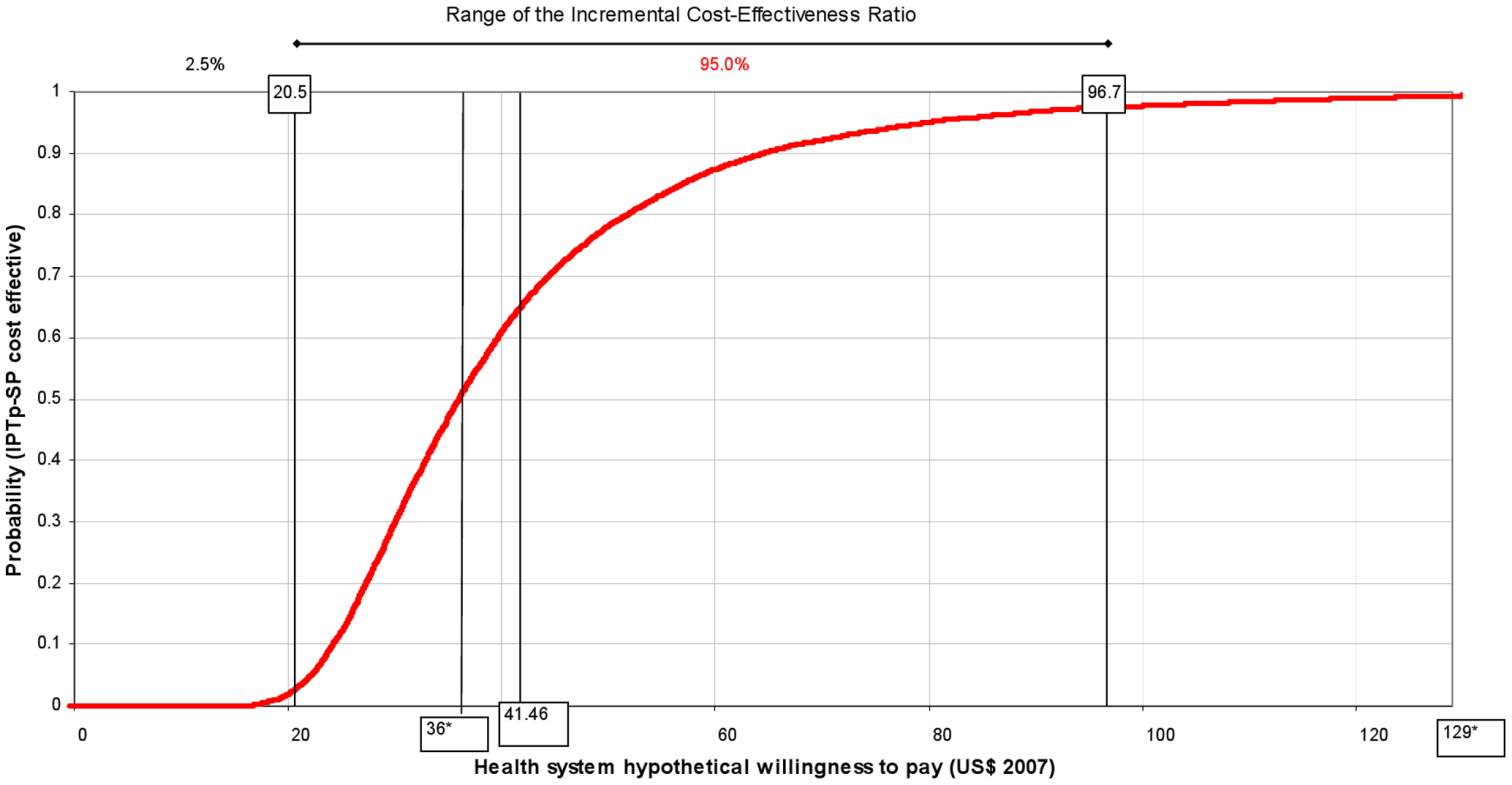
Maternal malaria: acceptability curve of the cost-effectiveness ratio of IPTp-SP^a^ vs hypothetical willingness to pay^b^. ^a^ Intermittent preventive treatment of malaria in pregnancy with sulphadoxine-pyrimethamine. ^b^ Acceptability curves were constructed by plotting the cumulative distribution of ICER of IPTp-SP per DALYs averted. The Y axis can be interpreted as probability that the intervention is cost-effective for every level of policy makers' ability or willingness to pay for each DALY averted (X axis). * 36 US$ per DALY averted = threshold of highly cost-effective intervention; 129 US$ per DALY averted = threshold of cost-effective intervention.

Per 1000 pregnant women receiving IPTp-SP, the number of outpatient episodes averted as a result of the intervention was 56.12 (CI 95% 20.50, 95.50) and the number of malaria admissions averted was 4.49 (CI 95% 1.63, 7.56). The number of DALYs averted was 12.20 (CI 95% 4.59, 20.81). Intervention costs per DALYs averted was 41.46 US$ (CI 95% 20.50, 96.70); its cumulative distribution (acceptability curve) is depicted in Figure 1.

Per 1000 women receiving IPTp-SP the total health system cost savings were 422.74 US$ (CI 95% 152.00, 718.00), 43% of which was due to a reduction in hospital admissions. Net intervention costs were 13.17 US$ (CI 95% −292.00, 290.00). With regard to household costs, for women attending as outpatients, 33.89 US$ (CI 95% 6.10, 77.20) were saved as direct costs and 83.79 US$ (CI 95% 29.60, 148.30) as indirect costs; for admitted women, 8.20 US$ (CI 95% −42.80, 55.80) were saved as direct costs and 11.44 US$ (CI 95% −20.50, 42.70) as indirect costs (Table 2).

**Figure 2:**
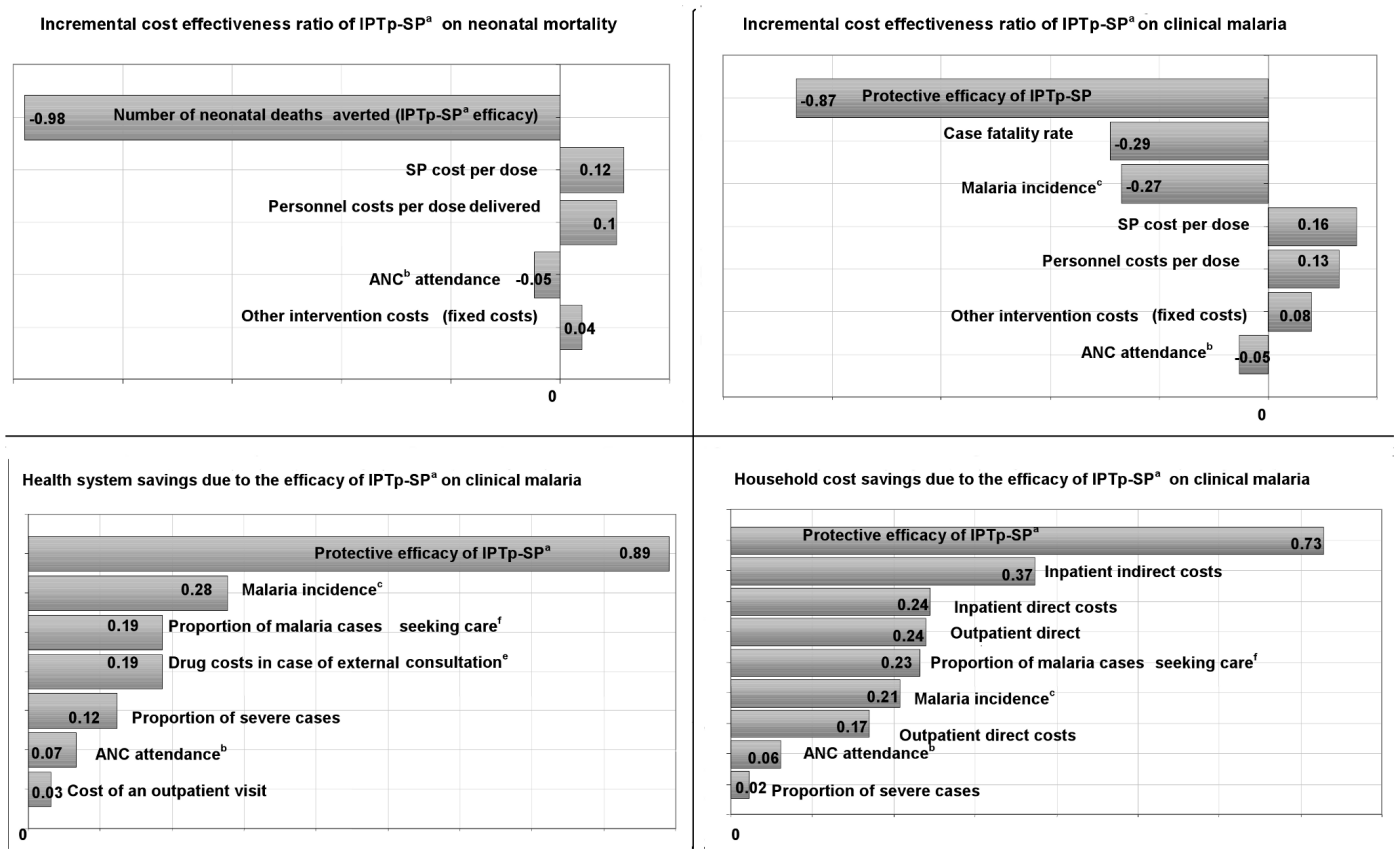
Correlation of cost-effectiveness ratios, savings, and input variables (Spearman's Rank). ^ a^ Intermittent preventive treatment of malaria in pregnancy with sulphadoxine-pyrimethamine.^ b^Antenatal Clinic (ANC) attendance at least twice during pregnancy. ^c^ Rate per person-year at risk in the placebo group. ^d^ Drug costs for inpatients refers to intravenous quinine.^ e^ Drug costs for outpatients are relative to artesunate plus SP. ^f^ It indicates the proportion of pregnant women with symptoms of malaria who seek formal health care.

Spearman's rank correlation coefficients showed that the PE of the intervention was the variable that most affected the ICERs in the estimated model (Figure 2). The associations between ICER and SP costs and between ICER and personnel costs were not strongly significant. The correlation coefficients between health system's savings and the PE of IPTp-SP showed a strong association. A similar association was found between the PE of IPTp-SP and households' savings.

**Figure 3:**
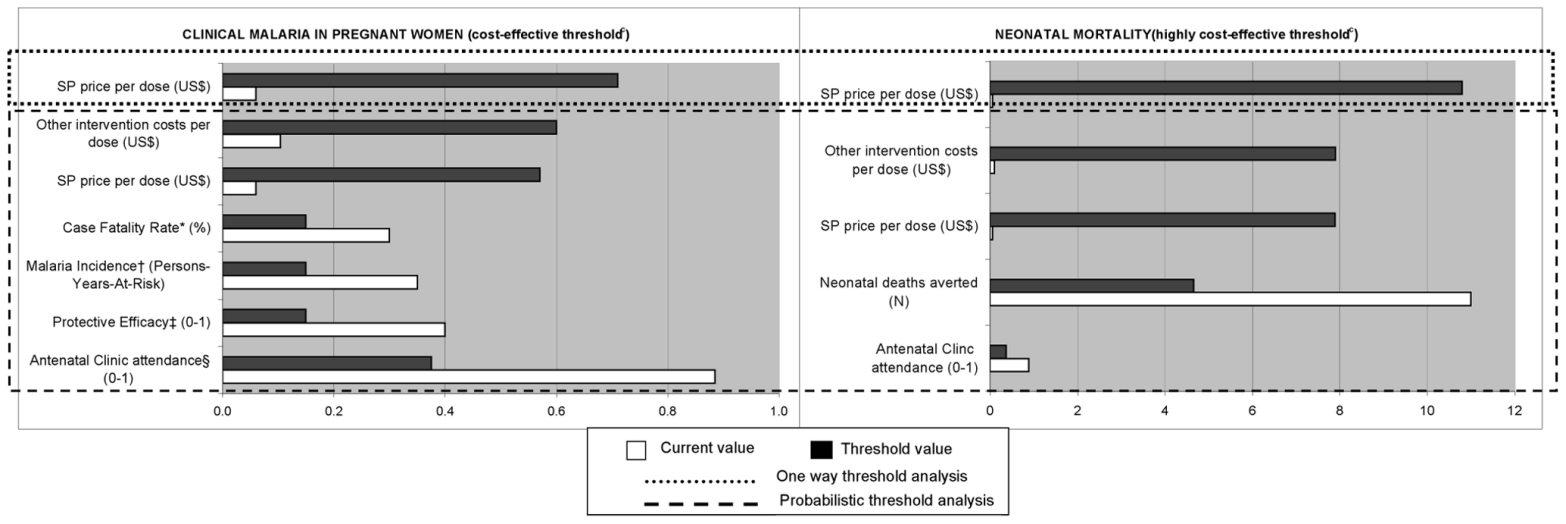
Threshold analysis of the cost-effectiveness of IPTp-SP^a^. ^a^ Intermittent preventive treatment of malaria in pregnancy with sulphadoxine-pyrimethamine ^b^ 129 US$/DALY averted. ^c^ 36 US$/DALY averted. Within the simulation ranges of each variable: * a threshold of 92.92 US$ was reached only. † a threshold of 96.79 US$ was reached only. ‡ a threshold of 85.99 US$ was reached only. ^ξ^ a threshold of 97.25 US$ was reached only.

According to the probabilistic threshold analysis of the ICER per DALY averted (Figure 3) IPTp-SP is no longer cost-effective when the ANC attendance is lower than 37.5%, PE is lower than 15%, malaria incidence is lower than 0.15 person-year at risk, case fatality rate lower than 0.15%. Furthermore, this analysis suggests that IPTp-SP is no longer cost-effective when the price of SP per dose is higher than 0.57 US$ and personnel cost for each dose delivered is higher than 0.60 US$. When all input variables of the cost-effectiveness analysis of IPTp-SP are constant (one way threshold analysis), the price of SP at which the intervention ceases to be cost-effective is higher (0.71 US$ per dose) than when the analysis is done through probabilistic analysis.

### Cost-effectiveness of IPTp on neonatal survival

**Figure 4:**
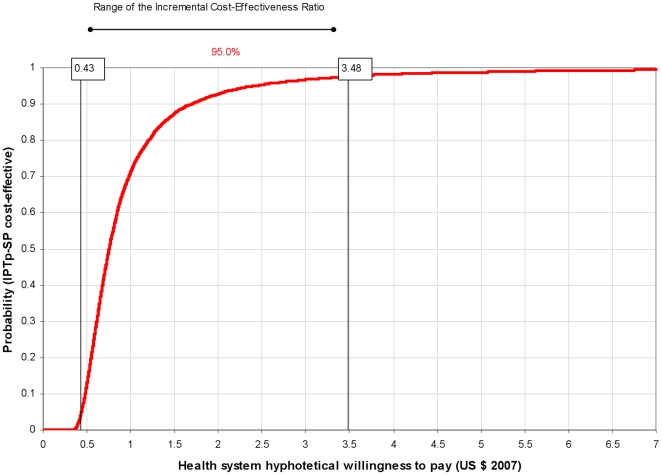
Neonatal mortality: acceptability curve of the cost-effectiveness ratio of IPTp-SP^a^ vs hypothetical willingness to pay^b^. ^a^ Intermittent preventive treatment of malaria in pregnancy with sulphadoxine-pyrimethamine. ^b^ Acceptability curves were constructed by plotting the cumulative distribution of ICER of IPTp-SP per DALYs averted. The Y axis can be interpreted as probability that the intervention is cost-effective for every level of policy makers' ability or willingness to pay for each DALY averted (X axis).

Delivering IPTp-SP to 1000 pregnant women translates into 18.93 (CI 95% 4.39, 33.85) neonatal deaths averted and into 555.21 (CI 95% 129.00, 992.00) DALYs averted (Table 2). The ICER was 1.08 US$ (CI 95% 0.43, 3.48) per DALY averted. The cumulative distribution of the ICER is presented in Figure 4.

Spearman's rank correlation coefficients showed that the PE of IPTp-SP is the variable having the greatest effect on the economic outcomes also in the case of neonatal mortality (Figure 2).

Figure 3 shows the threshold values of some variables beyond which IPTp-SP is no longer highly cost-effective in reducing neonatal mortality, but remains cost-effective. Such conditions, according to the probabilistic threshold analysis, are: ANC attendance lower than 37%; number of neonatal deaths prevented lower than 4.66; and SP costs as well as personnel costs higher than 7.90 US$ per dose delivered. One-way threshold analysis showed that IPTp-SP was still highly cost-effective at a SP price per dose of 11 US$.

## Discussion

The results of the cost-effectiveness evaluation of malaria prevention in pregnancy with IPTp-SP in this rural area of Mozambique showed that this intervention is highly cost-effective when compliance with ITNs use is high. The intervention was cost-effective even at the 95% confidence higher limits of the estimated ICERs. IPTp-SP is a very cheap prevention when provided through the ANC. Net intervention costs for 1000 pregnant women were 13.17 US$ (CI 95% -292.00, 290.00). Net intervention costs resulted negative in most of the left part of their distribution and, thus, IPTp-SP is likely to be a cost saving intervention. In a context of very limited economic resources for health care this is a very attractive intervention.

Parasite resistance to SP in the study area has been assessed *in vivo* in the case of treatment of symptomatic non complicated malaria in children [50]. However, there is no evidence of parasite resistance to SP when the drug is used for prevention in asymptomatic individuals, as in the case of pregnant women attending the ANC [51]. The reference trial for this economic evaluation assessed the efficacy of IPT-SP on malaria prevention during pregnancy, and the results showed that SP was highly efficacious in preventing clinical malaria 30 days after each administration [22]. However, drug resistance can evolve rapidly and a reduction in the efficacy of SP would worsen the cost-effectiveness of the intervention. In fact, the lower limit of protective efficacy allowing to define IPTp-SP as a cost-effective intervention against maternal malaria was shown to be of 15% in this study. Fifteen per cent is included in the confidence interval of IPTp-SP efficacy resulted from the trial [Protective Efficacy (PE) 40% (95% CI 7.4%, 61.2%); p = 0.020]. However, safety and efficacy of new drugs for IPTp have been already proved [52]. Any improvement in antimalarials efficacy would ameliorate the cost-effectiveness of the intervention despite an increase in its cost, within the boundaries estimated in this study. Further trials assessing the safety and efficacy of new antimalarials for IPTp are ongoing [15].

In the context of the trial the compliance of pregnant women with the use of ITNs was high and, thus, the cost-effectiveness of IPTp-SP assessed in this study assumes high ITNs coverage [22]. Currently, ITNs are provided for free as part of routine ANC services in Mozambique. However, their use outside a trial context should be encouraged to improve compliance at least during pregnancy and first years of life of the child.

Malaria infection significantly contributes to maternal morbidity in sub-Saharan Africa. A recent study in Manhiça showed that about a third of pregnant women attending the maternity clinic of the MDH presented a malaria clinical episode [43]. Maternal malaria also negatively affects neonatal survival [23]. Thus, interventions aimed to prevent these negative outcomes are of major public health relevance. However, a critical consideration when deciding whether an intervention should be implemented is its economic implications. Although it has received more attention recently, little information still exists on the cost-effectiveness of malaria preventive interventions in pregnancy. All published reports on the economic evaluation of preventive strategies for malaria in pregnancy used surrogate indicators of mortality to calculate DALYs [6], [7], [9] and only two cost-effectiveness analysis were carried out as part of intervention studies [5], [6]. One study was focused on the incremental cost-effectiveness analysis of administering IPTp-SP through community-based delivery compared to the delivery by means of the ANC and results are not comparable with the current study [5]. The other study, focused on the cost-effectiveness of alternative antimalarial regimens (chloroquine versus sulfadoxine-pyrimethamine), concluded that SP given during the second and beginning of the third trimester of pregnancy, was the most cost-effective option (75 US$ per infant death averted). In this study no savings due to the intervention were estimated, and infant deaths averted were extrapolated from the reduction of the low birth weight prevalence rather than being a direct consequence of the intervention.

A recent cost-effectiveness analysis of intermittent preventive treatment of malaria in infants (IPTi) with SP carried out in this same area of Manhiça, also found that the intervention was highly cost-effective in preventing malaria in infants [53]. It is important to point out that the delivery of both interventions through already existing health structures, such as the routine Expanded Program on Immunization (EPI) and the ANC is probably one of the most important factors for these strategies to be cost-effective.

In the current study, for DALYs calculation on maternal health it was assumed that reducing malaria morbidity would translate into fewer maternal deaths. To calculate the maternal mortality component of DALYs averted due to IPTp-SP administration, an average case fatality rate of 0.33% was applied. While there is insufficient information on how many infected pregnant women die of malaria in sub-Saharan Africa, the rate applied in this study appears to be realistic according to the available evidence. It was assumed that a third of pregnant women living in the study area were infected with malaria based on a recent report showing that 27% of pregnant women attending a health facility with clinical complaints suggestive of malaria were parasitemic [43]. The estimates used in this economic evaluation considered malaria as an important cause of maternal death in Mozambique as shown in a recent study on the causes of maternal mortality where malaria infection accounted for 10% of all maternal deaths in a tertiary hospital of Maputo [54].

With respect to DALYs calculation on neonatal health, this economic evaluation differs from previous ones in that mortality data, derived directly from the main trial, were used to calculate the mortality component of DALYs averted due to IPTp-SP administration, which gives more weight to the findings.

Mean values of the ICERs resulting from this study (41.46 and 1.08 US$) indicated cost-effective levels of investment for each DALY averted. In addition, acceptability curves (figures 1 and 4) helped defining which would be a cost-effective investment level per DALY averted according to the health system's willingness to pay. Figure 1 shows that if policy makers are willing to pay only 20 US$ or less per DALY averted, the probability that the intervention is cost-effective is close to 0. At a higher willingness to pay of 100 US$ per DALY averted the probability that IPTp-SP is cost effective is close to 100%. A willingness to pay of 6 US$ per DALY averted, in the case of neonatal mortality, would be high enough to guarantee at a probability of almost 100% that the intervention is cost-effective (Figure 4).

A recent economic evaluation estimated the cost-effectiveness of adding ITNs distribution through ANCs when antenatal services administer IPTp-SP [38]. Although results of this study and of the current one are not directly comparable, it may be useful to comment on the respective acceptability curves. In that study it was reported that with an investment of 15 US$ (2005 prices) per DALY averted, the probability of ITNs distribution to be cost-effective was of almost 50%, and with an investment of 106 US$ the probability would increase to 100%. In contrast, in the current study, 15 US$ per DALY averted would guarantee a 100% probability to be highly cost-effective in the case of neonatal survival, while 106 US$ per DALY would lead to a probability of about 100% that the intervention is cost-effective on maternal malaria.

Even if the Mozambican Government highly subsidies the cost of health care, being unhealthy has a significant negative economic impact for the households, especially due to the indirect costs of the illness. In this country, where the Gross Domestic Product (GDP) per capita in 2007 was 364 US$ (less than 1 US$ per person per day), the overall (including both direct and indirect component) cost for the household of an outpatient attendance or a hospital admission due to malaria, approximately equals the remuneration of 2 and 10 working days of a person working as peasant, respectively [55].

Health system and household savings from fewer neonatal deaths were not included in this economic evaluation. Health system savings estimation would imply a complex evaluation of the economic value of the failure of preventing a neonatal death. From the household point of view, avoiding a child death may have several social and familial repercussions apart from the direct monetary savings related to health care that are very difficult to estimate. An exhaustive economic evaluation would require a long term analysis focusing on behavioural changes that the death of a child could cause in the family as a whole, such as future fertility decisions and consequences on the social and familial role of the mother of the dead child, among others. It would be also necessary to include the impact of the decrease in neonatal mortality on economic growth. On one hand, a decrease in child mortality lowers the demand for children (demographic transition) and raises the income per capita. On the other hand, a decrease in child mortality provides an incentive to invest in education because of the higher return on investment consequent to the longer life expectancy the decrease in mortality leads to. Both the increase in per capita income and in education are essential premises towards economic growth [56], [57]. Nevertheless, it can be speculated that if such long term consequences were included, the intervention would be even more advantageous.

### Conclusions

IPTp-SP in the context of ITNs is highly cost-effective in Mozambique mainly because it is administered through already existing health structures, such as the ANC, and because SP is a cheap drug. IPTp-SP remains cost-effective even with a wide increase in both the cost of the drug and in other related intervention costs. These findings are likely to hold for other settings where IPTp-SP is implemented through routine ANC visits. The estimated ICERs leave wide space for all the input variables to change and for the intervention to remain cost-effective. The cost-effectiveness of IPTp would highly improve with an increase in the efficacy of the intervention. In areas such as Mozambique where malaria in pregnancy represents a major public health problem, investing in the improvement of the effectiveness of IPTp is highly recommended.
